# Effect of lead on root growth

**DOI:** 10.3389/fpls.2013.00175

**Published:** 2013-06-06

**Authors:** Mouna Fahr, Laurent Laplaze, Najib Bendaou, Valerie Hocher, Mohamed El Mzibri, Didier Bogusz, Abdelaziz Smouni

**Affiliations:** ^1^Laboratoire de Physiologie et Biotechnologie Végétale, Faculté des Sciences, Université Mohammed V - AgdalRabat, Morocco; ^2^Laboratoire de Biotechnologie des Plantes, Centre National de l’Energie, des Sciences et des Techniques Nucléaires, Unité de Biologie et Recherches Médicales- Division Sciences du VivantRabat, Morocco; ^3^Equipe Rhizogenèse, Institut de Recherche pour le Développement, Unité Mixte de Recherche Diversité Adaptation et Developpement des Plantes,Université Montpellier 2Montpellier, France; ^4^Laboratoire mixte international Adaptation des Plantes et microorganismes associés aux Stress Environnementaux, Laboratoire Commun de Microbiologie Institut de Recherche pour le Développement/Institut Sénégalais de Recherches Agricoles/Université Cheikh Anta Diop, Centre de Recherche de Bel AirDakar, Senegal

**Keywords:** root, lead, tolerance, uptake, root development

## Abstract

Lead (Pb) is one of the most widespread heavy metal contaminant in soils. It is highly toxic to living organisms. Pb has no biological function but can cause morphological, physiological, and biochemical dysfunctions in plants. Plants have developed a wide range of tolerance mechanisms that are activated in response to Pb exposure. Pb affects plants primarily through their root systems. Plant roots rapidly respond either (i) by the synthesis and deposition of callose, creating a barrier that stops Pb entering (ii) through the uptake of large amounts of Pb and its sequestration in the vacuole accompanied by changes in root growth and branching pattern or (iii) by its translocation to the aboveground parts of plant in the case of hyperaccumulators plants. Here we review the interactions of roots with the presence of Pb in the rhizosphere and the effect of Pb on the physiological and biochemical mechanisms of root development.

## INTRODUCTION

Lead (Pb) is a heavy metal of anthropogenic origin (Sharma and Dubey, 2005). Pb is a pollutant that accumulates in soils, sediments, and water and is extremely persistent in the environment (Traunfeld and Clement, 2001). Pb has no biological function and it is toxic to living organisms even at low concentrations. Although Pb is not an essential element, some plant species proliferate in Pb-contaminated area and accumulate it in different parts. Roots are the first organ in contact with the various components of rhizosphere (Lynch and Whipps, 1990). Roots have evolved various mechanisms to reduce Pb uptake and transfer to the aboveground parts of the plant, and limit its deleterious effects. This article reviews the origins of Pb contamination, availability, and uptake of Pb, and recent knowledge on physiological, biochemical, and ultrastructural changes in roots due to the presence of Pb in the rhizosphere.

## SOURCES OF LEAD

Lead is one of the most widely distributed trace metals. It is ranked second of all hazardous substances by the Agency for Toxic Substances and Disease Registry (ATSDR, 2007). Because of natural deposits and increasing human activities, Pb has become ubiquitous in the soil and in the environment. Natural inputs include weathering and erosion of parent rocks that Pb to the transfer of large quantities of metals to water bodies and land (Gadd, 2010). Volcanic eruptions also contribute to natural inputs. For instance, the atmospheric emission from volcanoes was estimated at 540–6,000 tons for 1983 (Nriagu and Pacyna, 1988), and 1,000–10,000 tons for 2001 (Richardson et al., 2001). Pb has been used by humans for centuries but anthropogenic activities related to this metal have increased significantly in recent decades. These activities include mining, smelting, fuel combustion, synthetic fertilizers, and various industrial processes: building construction, Pb-acid batteries, bullets and shot, solder, pewter, and fusible alloys (Mukai et al., 2001). Human activities significantly influence the global cycles of Pb. In 2004, 3,150,000 tons of Pb were extracted from the earth’s crust and brought into circulation in society (USGS, 2006). In 1983, a total of 400,000–1,000,000 tons of mobilized Pb were disposed of with waste from metal extraction (Nriagu and Pacyna, 1988).

Lead is not biodegradable and is extremely persistent in both water and soil. Pb can be retained in the environment for 150–5000 years (Saxena et al., 1999). Most of Pb accumulates in the top 8″ of the soil where it has a very low mobility. Without remedial action, high soil Pb levels will never return to normal (Traunfeld and Clement, 2001). When Pb enters the soil matrix, it is very difficult to remove it. The capacity of soil to adsorb Pb increases with increasing pH, cation exchange capacity, redox potential, organic carbon content, and chelates (phosphate) levels (United States Environmental Protection Agency, 1992).

The main part of the extracted Pb will not contribute to long-range environmental transport or be in a form that is readily bioavailable, but may later in the life cycle Pb to local impacts if not managed properly.

## AVAILABILITY AND UPTAKE OF LEAD BY ROOTS

The rhizosphere is where interactions take place between roots and soils constituents (Lynch and Whipps, 1990). When a root absorbs water or nutrients from soil, ions and molecules move toward this organ both by mass flow with soil water and by diffusion (Robinson, 1991). Pb may be present in different fractions in the soils. It was previously thought that Pb had low solubility and availability for plant uptake because it forms precipitates with phosphates, sulfates, and chemicals in the rhizosphere (Blaylock and Huang, 2000). These geo-chemical forms of Pb in soils affect its solubility, which directly influences its mobility. However, roots produce and excrete protons, exudates and several metabolites, which can modify the soil pH and thus interfere with the dissolution processes and formation of soluble metal–organic complexes (Leyval and Berthelin, 1991). Citric, fumaric, and uronic acids as well as many polysaccharides are able to form complexes and to chelate metal ions including Pb (Mench et al., 1987). Indeed, in *Vicia faba* and *Typha angustifolia*, Pb uptake by roots was shown to increase significantly in the first hour after adding organic ligands [ethylenediaminetetraacetic acid (EDTA), citric acid; Muhammad et al., 2009; Shahid et al., 2012]. However, Quartacci et al. (2006) reported that citric acid supplied to a metal contaminated soil did not cause any change in metal uptake in *Brassica juncea.*

Lead uptake is greatly affected by rhizospheric processes. Lin et al. (2004) explained the ability of *Oryza sativa* L. to absorb high levels of Pb from soil by a decrease in soil pH due to root exudates, solubilization of Pb by rhizosphere microorganisms and complexation of Pb with organic matter at the soil–root interface. These authors also found larger amounts of NH_4_OAc extractable Pb in the rhizosphere than in bulk soil, pointing to the involvement of root activities in changes in Pb availability (Lin et al., 2004).

Uptake of and tolerance to Pb depends on root system conditions. In sunflower, Pb accumulation and cell response was shown to differ between seedlings with a primary root system (PRS) and seedlings with adventitious root systems (ARS) only (in which the primary roots were cut off). The ARS was found to be more tolerant to Pb than the PRS in *Helianthus annuus* L. and *Allium cepa* (Michalak and Wierzbicka, 1998; Strubińska, and Hanaka, 2011). This suggests that ARS have additional mechanisms that protect them against Pb penetration and Pb-induced oxidative stress. However, these mechanisms are still unknown.

Rhizosphere organisms also affect the metals availability and speciation. In *Lantana camara*, Pb accumulation in roots increases in the presence of earthworms (*Pontoscolex corethrurus*; Jusselme et al., 2012). Similar trends were observed in the *Thlaspi caerulescens* rhizosphere of Pb-contaminated soil (Epelde et al., 2008). These authors found that microbial activity was stimulated by interaction between microorganisms and macroorganisms. The effect of earthworms on Pb uptake may be due to their impact on the distribution of soil microorganisms by providing suitable conditions for microbial growth (Brown, 1995) but the mechanisms involved are not clear.

Lead availability is also affected by the presence of other heavy metals. Orroñoa et al. (2012) reported that Pb availability was reduced when it was supplied with five heavy metals (Cd, Zn, Cr, Cu, and Ni) that have an antagonist effect. These authors also reported that, when Pb was supplied alone or in ternary combination (with Zn and Cu), its availability increased due to the antagonistic interaction between Cu and Zn, which made Pb more available for plant uptake.

The uptake of Pb is based mainly on the plant species and the interaction between roots (structures and synthesized exudates) and the rhizosphere (biochemical properties). Indeed, several factors must be taken into account when developing strategies for phytoremediation of Pb. Besides the organic and mineral composition of the soil and rhizopheric organisms and microorganisms, the ability of roots to modify the mobility and the bioavailability of Pb by changing rhizospheric conditions can significantly contributes to a successful phytoremediation program.

## ROOT DEFENSE AGAINST LEAD STRESS

**FIGURE 1:**
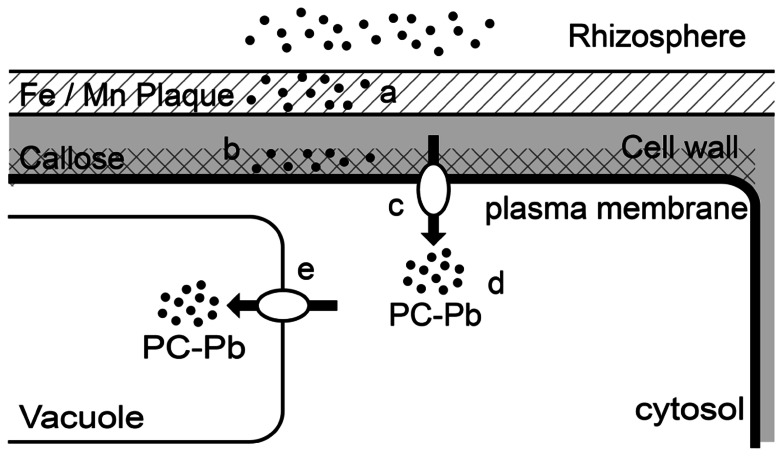
**Schematic representation of the types of root responses to lead toxicity in higher plants**. (a) Pb sequestration in the Fe/Mn plaques; (b) Pb binding in callose formed in the new cell wall; (c) Pb fluxes across the plasma membrane; (d) Chelation of Pb in the cytosol by phytochelatin; (e) Transport of PC–Pb complex and sequestration in the vacuole.

In response to Pb exposure, plants have developed a variety of tolerance mechanisms (**Figure 1**). Roots are the first organs, exposed to Pb ions (Piechalak et al., 2002). The first defense strategy is to stop the metal entering the root tissues by excluding it (Mishra et al., 2006). Roots rapidly respond to the presence of Pb by forming mechanical barrier. In some plants, there is synthesis and deposition of callose between the plasma membrane and the cell wall. This newly formed structure functions as a barrier against stress factors including metals (Bacic et al., 2009; Krzesłowska, 2011). Samardakiewicz et al. (2012) examined whether callose forms an efficient barrier against Pb penetration in the roots of *Lemna minor* L. exposed to 15 μM of Pb for 6 h. This treatment resulted in the synthesis and deposition of callose in the newly formed cell wall in the protoderm in the center of the root tip. After callose deposition the Pb concentration was restricted in these superficial cells. Similar observations have been made in other species exposed to Pb including *Arabidopsis thaliana* (Lummerzheim et al., 1995) and *Funaria hygrometrica* (Krzeslowska et al., 2009). Pb-induced callose deposition has been detected in the rhizodermis and in the center of the stele of Pb-treated soybean *Glycine max* roots tips (Samardakiewicz et al., 1996). Under metal stress, the synthesized callose inhibits cell-to-cell transport. This may result in the prevention of a wide incursion of Pb ions, but it can simultaneously inhibit the transport of other molecules. However, the synthesis of callose is not a general pattern in plants in response to Pb, in *Zea mays* and *G. max*, low level Pb treatment did not result in any callose deposition in root tissue. Although, these species synthesized callose in response to cadmium or arsenic (Pirselova et al., 2012). It seems that the formation of callose was closely related to the amount of Pb entering the cell, and subsequently the level of stress.

In some plants, the formation of Fe and Mn plaques on roots surface may provide a means of attenuation and external exclusion of metals. These plaques increase the sequestration of Pb on the root surface and in the rhizosphere, providing a means of external exclusion of soil Pb (Hansel et al., 2002). In some rice cultivars, Fe plaques were shown to affect patterns of Pb uptake and accumulation. Lower concentrations of Pb were found in the root tissues of rice plants with plaque compared to concentrations found in the plants without plaque. But the functions of plaque are limited as they are only efficient in relatively low or moderately Pb-contaminated soil (Liu et al., 2011). Fe plaque and organic matrix with high Pb affinity were found in root epidermis of *Typha latifolia*, and were shown to prevent the accumulation and the translocation of Pb within the root (Qian et al., 2012; Feng et al., 2013).

In most plants, 90% of the total Pb is accumulated in roots (Kumar et al., 1995). Most Pb in roots is localized in the insoluble fraction of cell walls and nuclei, which is linked with the detoxification mechanism (Piechalak et al., 2002). After exposure to Pb, cell mechanisms that minimize the potential for toxicity are rapidly activated. In the roots of several species including *Pisum sativum* (Malecka et al., 2009); *Allium sativum* (Jiang and Liu, 2010), and *Athyrium yokoscense* (Nishizono et al., 1987), the cell walls, the first barrier against Pb stress, can immobilize and accumulate some or even most Pb ions. The important role of the cell wall in the defense response of plants to trace metals was recently reviewed by Krzeslowska (2011). The capacity of cell walls to bind divalent metal cations mainly depends on the amount of polysaccharides with many carboxyl groups (Inoue et al., 2013). In *Arabidopsis thaliana*, Pb-galacturonic acid fragments were detected in root treated with Pb (Polec-Pawlak et al., 2007). Brunet et al. (2008) showed that root of *Lathyrus sativus *L*.* exposed to Pb contained much less calcium than control plants, and explained the reduction in Ca content by the replacement of Ca ions by Pb ions, which have a high affinity for pectin in cell walls. In *Raphanus sativus*, Pb^2^^+^ was also shown to bind to carboxyl groups of pectin in cell walls (Inoue et al., 2013). All the examples described above clearly show that the cell wall is one of the preferred and essential compartments for Pb accumulation, deposition, and sequestration. Therefore, these results shed a new light on the functioning of the cell walls in plant cell defense strategy against Pb. Heavy metals including Pb are likely to enter plant cells via essentials cations transporters. *AtCNGC*, homologous to a non-selective cation channel, was suggested to enable Pb^2^^+^ entry since overexpression of the truncated gene resulted in tolerance to Pb^2^^+^ (Sunkar et al., 2000). Ca^2^^+^ was also reported to compete with Pb^2^^+^ for entry into rice root cells. When Ca^2^^+^ was supplied in the medium, it reduced Pb uptake and toxicity (Kim et al., 2002). This suggests that Pb enters the root cells via Ca^2^^+^/Mg^2^^+^ gated channel (Kim et al., 2002).

In *Allium sativum*, as soon as excessive Pb ions enter the cytoplasm, a defense mechanism is activated, protecting the cells against Pb toxicity. Endocytotic and exocytotic processes are involved in these phenomena. The plasma membrane represents a “living” barrier of the cell to free inward diffusion of Pb ions. Invaginations of plasmalemma and some vesicles from dictyosomes and endoplasmic reticulum (ER) could prevent the free circulation of Pb ions in the cytoplasm. The vacuole is ultimately one of the main storage sites for metal sequestration (reviewed by Sharma and Dubey, 2005; Clemens, 2006). In *Allium sativum* roots, cysteine-rich peptides commonly referred as phytochelatins (PCs) were detected only after 2 h of Pb exposure (Jiang and Liu, 2010). This indicates that Pb ions can induce synthesis of PCs. Piechalak et al. (2002) demonstrated that the synthesis of PCs takes place under the influence of Pb ions in root cells of three tested plant species of the Fabaceae family: *Pisum sativum*, *V. faba*, and *Phaseolus vulgaris*. The complex PC–Pb formed is then transported through the cytosol into the vacuoles (Piechalak et al., 2002). *AtHMA3*, encoding a *P *_ 1B-2_-ATPase , a heavy metal transporter, is localized in the vacuolar membrane of roots cells in *Arabidopsis thaliana* (Talke et al., 2006; Morel et al., 2009). This transporter is involved in the transfer of complexed heavy metals, including Pb, from the cytoplasm to the vacuole (Morel et al., 2009). Root length was less affected by Pb in *Arabidopsis thaliana* plants overexpressing *AtHMA3* than in wild-type plants (Morel et al., 2009). *B. juncea* appears to tolerate high concentrations of Pb thanks to its efficient cell roots vacuolar storage mechanisms. In this species, Pb sequestration was restricted to vacuoles (Meyers et al., 2008). In addition, it was suggested that exposure to Pb causes the production of additional vacuole specifically for Pb storage in the root tips of *B. juncea* (Meyers et al., 2008). The increase in the production of vacuoles could be regarded as a defense and adaptation strategy to elevated levels of Pb in the root cells. This roots potential storage can be used in phytoremediation processes. **Table 1** shows a list of plant species effective in the accumulation of Pb in roots that could be used in rhizoremediation.

**Table 1:** Plant species proposed for lead rhizoremediation.

Plant species	Area of application	Reference
*Carex pendula*	Wastewater	Yadav et al. (2011)
*Pistia stratiotes*	Contaminated water	Baharudin (2008); Vesely et al. (2012)
*Eichhornia crassipes*	Aquatic system	Tiwari et al. (2007)
*Scirpus americanus*	Aquatic system	Santos-Dïaz and Barrón-Cruz (2011)
*Phaseolus vulgaris*	Contaminated water	Piechalak et al. (2002)
*Typha latifolia*	Aquatic system	Santos-Dïaz and Barrón-Cruz (2011)
*Cistus libanotis*	Contaminated soil	Laplaze et al. (2010)
*Hirschfeldia incana*	Contaminated soil	Auguy et al. (2013)

In a metallicolous ecotype of *Elsholtzia argyi*, Pb is found in fine particles dispersed through root cell membranes and cell wall fractions whereas in non-metallicolous roots, most Pb was found as large aggregates deposited in the cell wall fractions. These differences in localization explained why non-metallicolous roots were not able to transfer Pb to above ground parts via the apoplast (Islam et al., 2007). In some plants, Pb can be transported *via* vascular tissues to aerial parts (Hanc et al., 2009). In *Sesbania drummondii*, Pb is transported to leaves after complexation with acetate, nitrate, and sulfide (Sharma et al., 2004). In tobacco, a *cyclic nucleotide gated channel* (*NtCBP4*) was suggested to be involved in Pb transport (Sunkar et al., 2000).

To sum up, Pb pathway may include the following stages in roots: Pb can bound with physical barrier (callose, Fe/Mn plaques, cell wall...). At high concentration, this barrier is broken and the flux of Pb enters the cell through the plasma membrane using the ions transporters. In cytoplasm, Pb is chelated with PCs. The complexe formed is then sequestered in the vacuoles. In accumulator plants, Pb can be transported via phloem to aerial parts (**Figure 1**).

Compared to Zn and Cd, very little is known about the molecular mechanisms of acquisition, transport, and accumulation of Pb. This is due first to the characteristics of Pb which precipitates with some components of the culture media making difficult to study its bioavailability to the roots. On the other hand, the lack of model plant for studying the mechanisms of tolerance to this metal. Among the 450 species known as metal hyperaccumulator and tolerant plants, Pb accumulating species are rather exceptional. Recently, Auguy et al. (2013) identified *Hirschfeldia incana*, a member of the Brassicaceae family, as a Pb accumulator plant. They demonstrated that this species, owing to its close genetic proximity to *Arabidopsis*, is a good model to identify genes involved in Pb tolerance and accumulation. This can open up new possibilities for understanding the molecular mechanisms of Pb tolerance in plants.

## EFFECT OF LEAD ON ROOT DEVELOPMENT AND PHYSIOLOGY

### PHYSIOLOGY AND ULTRASTRUCTURAL EFFECTS OF LEAD

The primary effect of Pb toxicity in plants is a rapid inhibition of root growth, probably due to the inhibition of cell division in the root tip (Eun et al., 2000). It was demonstrated that Pb caused inhibition of cell division in *Lemna minor* roots (Samardakiewicz and Wozny, 2005). In several plant species, including *Triticum aestivum* (Dey et al., 2007; Kaur et al., 2013), *Z. mays* L. (Kozhevnikova et al., 2009), *Pisum sativum* (Malecka et al., 2009), and *Sedum alfredii* (Gupta et al., 2010), a decrease in the length and in root dry mass under Pb toxicity have been reported (Munzuroglu and Geckil, 2002). Verma and Dubey (2003) showed that growth of rice roots was significantly inhibited at 0.5–1 mM Pb^2^^+^; up to 40% reduction in root length was observed in 20-day-old rice seedlings. In Pb-treated *Elsholtzia argyi* and *Elsholtzia splendens*, the length and surface area of roots were strongly affected (Peng et al., 2005).

In response to Pb exposure, roots can also respond via changes in volume and diameter, with the production or inhibition of lateral roots. Root cells viability in rice is affected by Pb^2^^+^ ions and cell death increased at different Pb concentrations (Huang and Huang, 2008). Furthermore, cell wall distention, formation of folds, protuberances, and nicks were observed in response to different Pb concentrations in *Triticum aestivum* (Kaur et al., 2013), *Elsholtzia argyi* (Islam et al., 2007), and *Allium cepa* (Wierzbicka, 1998). Pb has been reported to disrupt microfibrils and microtubules, resulting in the formation of folds (Liu et al., 2009). In addition, Kaur et al. (2013) observed distentions and lesions in cell wall of *Triticum aestivum* roots as a result of activation of certain wall-degrading enzymes in response to Pb exposure. In *Z. mays* roots, Pb treatment resulted in Pb accumulation in the meristem in both apoplastic and symplastic pathways, associated with changes in microtubule organization (Eun et al., 2000).

Lead also has an impact on mineral homeostasis. Brunet et al. (2008) found that roots of *Lathyrus sativus* exposed to Pb showed an increase in Pb content along with an increase in Na levels, which is absorbed to compensate the loss in K. A reduction in Ca contents in Pb-exposed plants has also been observed in other species, such as maize, tomato, and mustard varieties (White and Broadley, 2003; Sharma and Dubey, 2005) and could result from the inhibition of Ca transporters by Pb ions (Wojas et al., 2007) and/or replacement of Ca ions with Pb ions due to its high affinity for Ca binding-sites on biological structures (Habermann et al., 1983). A reduction in Zn, Cu, and K contents in response to Pb exposure was observed in *Cucumis sativus* and *Z. mays* plants, as a result of a possible blockage of the transporter proteins by Pb (Sharma and Dubey, 2005).

Finally, Pb induces genotoxicity in plants (Rucińska et al., 2004). The comet assay evaluating the DNA-damaging effect of Pb showed an increase in DNA damage in root nuclei of tobacco and lupin (Rucińska et al., 2004; Gichner et al., 2008).

### BIOCHEMICAL EFFECTS OF LEAD

 The cytotoxic mechanisms of Pb in plants are not entirely understood. It has been reported that Pb leads to the overproduction of reactive oxygen species (ROS) such as superoxide radicals (radical O^2^^-^) and hydrogen peroxide (H_2_O_2_) in plant cells (Reddy et al., 2005; Liu et al., 2010). These can cause lipid peroxidation, membrane damages, and oxidative stress (Sharma and Dietz, 2009). When pea (*Pisum sativum*) roots were exposed to 0.1 and 0.5 mM of Pb(NO_3_)_2_, a rapid increase in superoxide anion ($O_{2}^{-}$) and H_2_O_2_ levels occurs after 2 and 8 h of Pb treatment, respectively (Malecka et al., 2009). Liu et al. (2012) reported that after Pb treatment, roots of *Ficus microcarpa* produced high concentrations of H_2_O_2_ along with an increase in $O_{2}^{-}$ accumulation. $O_{2}^{-}$ is produced by nicotinamide adenine dinucleotide phosphate (NADPH) oxidase in the plasma membrane, and is converted to H_2_O_2_ through non-enzymatic pathways or by superoxide dismutase (SOD; Passardi et al., 2004). Some ROS can alter gene expression and modulate the activity of specific proteins in the plant defense system (Sharma and Dubey, 2005). To protect cells and tissues from injury and dysfunction, plants have developed various strategies, such as over expression of SOD, catalase (CAT), peroxidase (POX), and ascorbate POX genes. In addition, non-enzymatic antioxidants with low molecular weights, such as proline, cysteine, non-protein thiol, ascorbic acid, and glutathione, which can reduce oxidative stress by scavenging ROS are synthesized (Choudhury and Panda, 2005; Singh et al., 2006; Malecka et al., 2009). Responses to metal toxicity involving these enzymes and non-enzymatic antioxidants differ depending on the plant species, type of tissue, and metal concerned. Huang and Huang (2008) showed that in rice roots, Pb^2^^+^-induced ROS production and Ca^2^^+^ accumulation and activated MAP (mitogen-activated protein) kinases (proteins kinase cascade and major pathways by which extracellular stimuli are transduced into intracellular responses in all eukaryotic cells; Jonak et al., 2002) which are located in the apical region in rice roots. They demonstrated that treatment with glutathione, a powerful antioxidant, decreased Pb^2^^+^-induced root cells death and reduced MAP kinases activity. An increase in H_2_O_2_ content upon Pb exposure was observed in response to Pb^2^^+^, with an increase in CAT activity in *Triticum aestivum* (Kaur et al., 2013), *Elsholtzia argyi* (Islam et al., 2007), and *Pisum sativum* (Malecka et al., 2009). Pb-induced lipid peroxidation and enhanced H_2_O_2_ content in roots of *Allium sativum* (Liu et al., 2009), *Z. mays* (Gupta et al., 2009), and *B. campestris* (Singh et al., 2011). However, Islam et al. (2007) and Kaur et al. (2013) reported a decline in the activity of POXs in *Elsholtzia argyi* and *Triticum aestivum* roots upon Pb exposure. Therefore, a higher concentration of Pb or longer treatment inhibit cell metabolism and H_2_O_2_ production, resulting in a decrease in the activity of some antioxidant enzymes (CAT; Verma and Dubey, 2003; Malecka et al., 2009). Plant enzymatic antioxidant defense systems vary with the plant species and with the intensity of Pb treatment. Production of ROS is common to different plant species. Some of these produced ROS may function as important signaling molecules by altering gene expression and modulating activity of specific defense proteins. However, all ROS can be very harmful to organisms at high concentrations.

Lead also increased protein and proline contents in roots of two varieties of *B. napus* with an increase in the concentration of Pb in the nutrient solution (Gohari et al., 2012). Proline plays an essential role in reducing environmental stress, including that caused by heavy metals. Piechalak et al. (2002) found that *V. faba* and *Pisum sativum* roots produced high amount of thiol peptides and PCs after Pb exposure. Although, the high level of these proteins allows tolerance to Pb for these species roots.

## CONCLUSION

Root system is the first organ in contact with the different components of the soil and water. By their exudates and their effects on rhizosphere activities (proliferation of microorganisms, metal chelation, acidification, etc.) plant roots can tolerate and in some cases accumulate high levels of Pb. An overall higher rate of accumulation was observed in roots rather than leaves in several species. Almost 90% of Pb accumulated in a number of species of the Brassicaceae family (Kumar et al., 1995) and some crops species such as *Z. mays* (Małkowski et al., 2002) and *Pistia stratiotes* (Vesely et al., 2012) was located in roots. This accumulator potential can be used in phytoremediation process. Rhizofiltration is a subset technique that uses both terrestrial and aquatic plants roots to absorb, concentrate, and precipitate metals from polluted water to their biomass (Dushenkov et al., 1995). This technique is cost-effective, and can be used for site restoration including maintenance of the biological activities of the polluted site. In this context, several plants have been identified whose roots could be used to clean up land contaminated by Pb. Therefore, improvement of the capacity of plant roots to tolerate and accumulate Pb by genetic engineering should open up new opportunities for rhizoremediation.

## Conflict of Interest Statement

The authors declare that the research was conducted in the absence of any commercial or financial relationships that could be construed as a potential conflict of interest.
